# Low concentrations of *Lactobacillus rhamnosus GG* (Yoba^®^) are safe in male *Drosophila melanogaster*

**DOI:** 10.1186/s13104-019-4297-x

**Published:** 2019-05-14

**Authors:** Keneth Iceland Kasozi, Aisha Bukenya, Ejike Daniel Eze, Josephine Kasolo, Dickson Stuart Tayebwa, Fred Ssempijja, Joy Suubo, Andrew Tamale, Isaac Echoru, Ibrahim Ntulume, Sarah Kemuma Onkoba, Lisa Nkatha Micheni, Emmanuel Tiyo Ayikobua, Oscar Hilary Asiimwe, Muhamudu Kalange

**Affiliations:** 1 Department of Physiology, Faculty of Biomedical Sciences, Kampala International University Western Campus, Box 71, Bushenyi, Uganda; 2 School of Pharmacy, Kampala International University Western Campus, Box 71, Bushenyi, Uganda; 3 Department of Biomedical Sciences, School of Medicine, Kabale University, Kabale, Uganda; 4 Department of Physiology, College of Health Sciences, Makerere University, Box 7062, Kampala, Uganda; 5 College of Veterinary Medicine Animal Resources and Biosecurity, Makerere University, Box 7062, Kampala, Uganda; 6 Department of Anatomy, Faculty of Biomedical Sciences, Kampala International University Western Campus, Box 71, Bushenyi, Uganda; 7 Department of Microbiology, Faculty of Biomedical Sciences, Kampala International University Western Campus, Box 71, Bushenyi, Uganda; 8 Department of Biochemistry, Faculty of Biomedical Sciences, Kampala International University Western Campus, Box 71, Bushenyi, Uganda

**Keywords:** Probiotics, Yoba for life, Nutritional toxicology, Yoghurt, Food supplements, Catalase, Hydrogen peroxide scavenging, Aging

## Abstract

**Objective:**

The purpose of the study was to generate information on the safety of probiotics, thus the study objectives were to evaluate the effects of Yoba^®^ on basic physiochemical properties. The study assessed male w^*1118*^ *Drosophila melanogaster* which were provided food supplemented with Yoba^®^ at 1%, 3%, 6%, and 12% on motor function, total protein, catalase activity, and hydrogen peroxide scavenging activity and lifespan.

**Results:**

Yoba^®^ at high concentration (≥ 6%) increased locomotor activity in *Drosophila melanogaster*, however, total protein, catalase, and hydrogen peroxide scavenging activity were significantly higher at 1% Yoba^®^ compared to 3%, 6%, and 12% Yoba^®^. Yoba consumed at 1% was associated with greater physiological benefits in *Drosophila melanogaster*. Findings in the study offer a rationale for the consumption of Yoba^®^ at 1% in humans as is currently being promoted by the Yoba for Life consortium, however, high concentrations of Yoba^®^ would disrupt physiological function as shown by this study.

## Introduction

Probiotics are micro-organism commonly consumed by both humans and animals [1, 2]. This is important since probiotics have established benefits such as up-regulation of immune-modulatory genes [3], modification of gastrointestinal ecology [4, 5], weight loss in obesity [6, 7], reduction of gastrointestinal pH, reduction of pathogenic bacteria and modification of host immune system [8, 9]. Despite these benefits of probiotics, there continues to be a scarcity of information on their safety due to conflicting reports on probiotic efficacy [8, 10–12]. *Lactobacillus rhamnosus GG* strain Yoba^®^ is an extensively studied probiotic which has gained rapid international consumption [13–15]. *Drosophila melanogaster* has been shown to be a reliable model in the evaluation of physiological and biochemical processes [16, 17]. Motor control in flying insects is essential for feeding, walking, mating and flying in addition to other advanced behaviors [18, 19], just as it serves to human through the involvement of the skeletomuscular system. Since chronic consumption of *Lactobacillus plantarum* TWK10 (a probiotic) has been associated with improved exercise and muscle mass in mice [20], effects of Yoba^®^ on physiological variables would be important to guide consumers on its safety. In *Drosophila melanogaster*, the N-termini proteins play a crucial role in disease following acetylation, myristoylation, and acylation [21]. This is important since the N-terminal glycine residue of target proteins, catalyzed by *N*-myristoyltransferase have been associated with colon cancers where they are upregulated in the early stages of tumor development in humans [22]. In *Drosophila melanogaster* dietary protein content has also been found to affect lifespan [23], and this would be increased following a heavy probiotic diet. Furthermore, increased expression of antioxidant enzymes (superoxide dismutase) has not been associated with an extension in lifespan in long-lived but would probably affect short-lived *Drosophila melanogaster* [24]. Controversy on these observations continues to grow [25], however, an extension in lifespan in mouse has been reported [26]. In addition, catalase has been associated with an increase in resistance to oxidative stress [27], while glutathione reductase increases lifespan at increased levels of oxidative stress, demonstrating the importance of antioxidant enzymes in *Drosophila melanogaster* [28]. Bearing in mind that consumption of probiotics has been associated with improved human health [9], the objective of the study was to evaluate the effects of Yoba^®^ on motor activity, catalase, hydrogen peroxide scavenging activity and lifespan in male w^*1118*^ *Drosophila melanogaster*.

## Main text

### Methods

Yoba^®^ was procured from a Yoba for Life^®^ distributor in Mbarara district of Uganda under the global distribution organization from the Netherlands [11]. Since 1 g of Yoba^®^ contains 10,000 *Lactobacillus rhamnosus GG* [13–15] and a dilution of 1% w/v is recommended for Yoghurt preparation for human consumption [14], experimental food was made of 1%, 3%, 6% and 12% w/v Yoba^®^ using this rationale. *Drosophila melanogaster*, *w*^*1118*^ stocks originally from National Species Stock Center (Bowling Green, OH, USA) at the Institute of Biomedical Research Laboratory of Kampala International University Western Campus were used as previously described [16]. Flies were exposed to the experimental diets for a period of 2 weeks (for sub-chronic toxicity) with 12 h of light and 12 h of darkness, and the following experiments were carried out.

#### Negative geotaxis

This was done using standard methods [16]. Flies were separated from their experimental diets and transferred into cylindrical calibrated testing vials with one open end. In the calibrated tube to indicate the 4 cm [29] and 8 cm [30], flies which had been immobilized by allowing them to settle at the bottom of the tube were allowed to move upwards (negative geotaxis), and the number of flies which crossed the marked distances in 10 s were simultaneously counted. Experiments were repeated 3 times for each vial with an allowable rest period of 1 min for each experiment thus, the number of flies used in this experiment were 5 vials × 10 flies × 5 groups = 250 flies.

#### Determination of catalase, total protein, and hydrogen peroxide scavenging activity

*Drosophila melanogaster* from individual exposure groups were anesthetized on ice and homogenized in 0.1 M sodium phosphate buffer, pH 7.0 (1 mg:10 μL), and centrifuged at 4000*g* for 10 min at 4 °C in a Mikro 220R centrifuge (Tuttlingen, Germany) as described previously [16]. Subsequently, the supernatant was separated from the pellet into labeled Eppendorf tubes and used for the determination of the activities of catalase, total protein and hydrogen peroxide scavenging activity. Thus, the number of flies used in this experiment were 5 vials × 10 × 5 groups × 3 experiments = 750 flies.

Catalase activity was determined according to standard methods [31]. A calibration curve was generated in the form y = mx + c using standard catalase concentrations for which the corresponding foam heights were determined i.e.

$${\text{Foam}}\;{\text{height}} ( {\text{y}}) = 0.056*{\text{Catalase}}\;{\text{activity}}\left( {\text{x}} \right) + 0.1,\;R^{2} = 0.9604.$$

Total protein concentrations of the various samples were determined as previously described [32] using a Eurochem^®^ total protein test kit according to the manufacturer’s recommendations.

The ability of *Drosophila melanogaster* to scavenge hydrogen peroxide was ascertained as previously determined [16].

#### Lifespan assay

This was done using standard methods using male *Drosophila melanogaster* [16, 17]. The number of flies used in this experiment were 5 vials × 10 flies × 5 groups = 250 flies. Fly food was changed two times per week for the duration of the experiment.

#### Data analysis

This was done using Graph Pad Prism Version 6 and information was presented as figures and a Table. ANOVA test was done and Tukey’s test was used to determine sources of variation and significant differences (P < 0.05) were indicated with different superscripts i.e. letters a, b, c. The lifespan data were analyzed using Kaplan–Meier survival analysis and Mantel-Cox was performed on the survival curves, with significance being reported when P < 0.05.

### Results

#### Yoba^®^ improved the climbing activity and hydrogen peroxide scavenging of male *W*^*1118*^*Drosophila melanogaster*

**Fig. 1:**
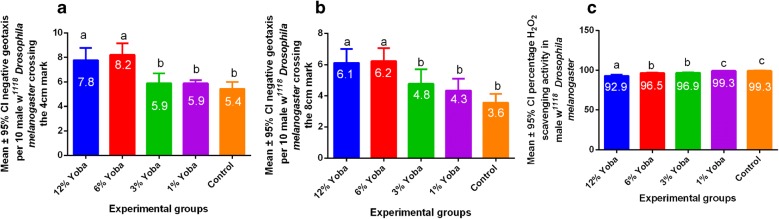
Locomotor activity associated with Yoba at varying concentrations in male *Drosophila melanogaster*

Locomotor activity was significantly higher (P < 0.05) in the 12% and 6% Yoba^®^ concentrations when compared to 3%, 1% and the control at the 4 cm and 8 cm mark in male *Drosophila melanogaster* respectively as shown in Fig. 1a, b and Table 1. In addition, no significant differences were observed between 12 and 6% as well as 3%, 1% and the control (Table 1). Furthermore, hydrogen peroxide scavenging activity was highest at 1% Yoba^®^ concentration as shown in Fig. 1c and Table 1.

**Table 1:** Multiple comparisons showing P values for negative geotaxis, total protein, catalase, hydrogen peroxide scavenging activity and lifespan in male *Drosophila melanogaster* after Yoba^®^ exposure

Tukey’s multiple comparisons tests	N	Negative geotaxis		Total protein	Catalase	Hydrogen peroxide scavenging	Lifespan
		4 cm	8 cm				
		Adjusted P values					
12% Yoba vs. 6% Yoba	50	0.874	0.9994	0.789	0.0784	< 0.0001	Log-rank (Mantel-Cox) test for curve comparisons *X*^2^ (4) = 18.52, P = 0.001
12% Yoba vs. 3% Yoba	50	0.002	0.0733	0.3771	0.0004	< 0.0001	
12% Yoba vs. 1% Yoba	50	0.002	0.0076	< 0.0001	< 0.0001	< 0.0001	
12% Yoba vs. control	50	0.0001	< 0.0001	0.0002	0.0006	< 0.0001	
6% Yoba vs. 3% Yoba	50	0.0001	0.0435	0.9578	0.0279	0.9192	
6% Yoba vs. 1% Yoba	50	0.0001	0.004	< 0.0001	0.0015	< 0.0001	
6% Yoba vs. control	50	< 0.0001	< 0.0001	0.0059	0.0468	< 0.0001	
3% Yoba vs. 1% Yoba	50	> 0.9999	0.8964	< 0.0001	0.3316	0.0006	
3% Yoba vs. control	50	0.874	0.1191	0.034	0.9969	0.0007	
1% Yoba vs. control	50	0.874	0.5252	0.0005	0.2114	> 0.9999	

*N* number of flies in each group

#### Yoba^®^ increased total protein, catalase activity and lifespan at low concentrations in male w^*1118*^*Drosophila melanogaster*

**Fig. 2:**
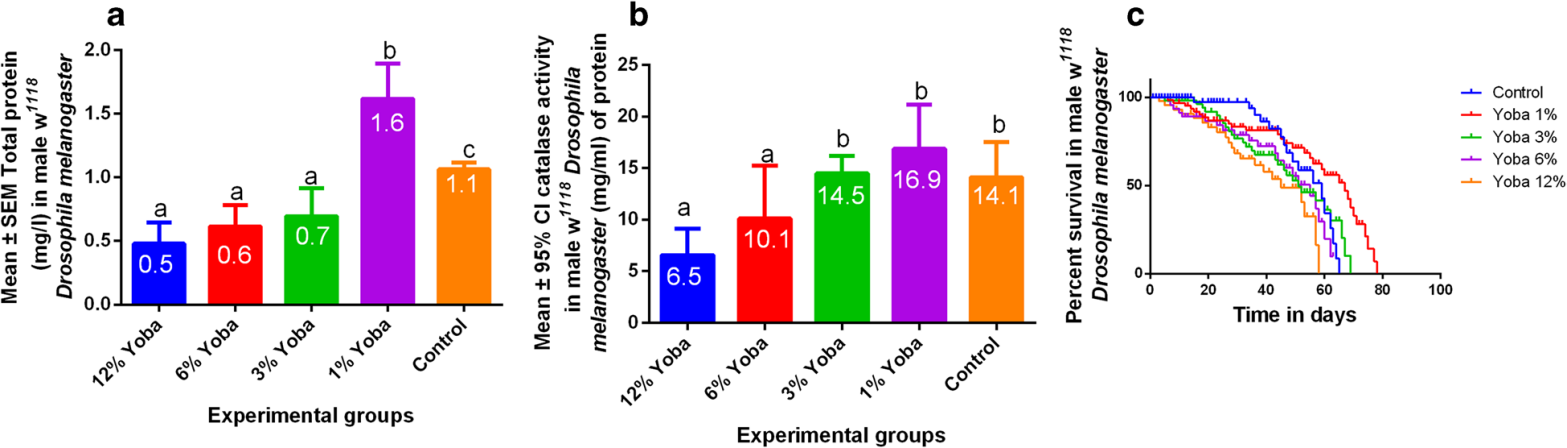
Changes in activity in *Drosophila melanogaster* following Yoba exposure. **a** Total protein levels; **b** catalase activity, **c** lifespan

Total protein levels were significantly higher at 1% Yoba^®^ concentration and lower at higher Yoba^®^ concentrations, as shown in Fig. 2a and Table 1. Catalase activity was also found to be significantly highest in the order of 1% > 3% > control > 6% > 12% Yoba^®^ concentration (Fig. 2b and Table 1). In the first 20 days, survival was lowest in the Yoba^®^ exposed groups, however, this changed after day 50 at which survival was highest in the 1% Yoba^®^ group. By day 60, all flies from the 12% Yoba^®^ group had died while by day 70, no fruit flies from the control and 6% were still surviving. *Drosophila melanogaster* survival rate was significantly highest in the order of 1% > 3% > 6% > 12% as shown in Fig. 2c and Table 1.

### Discussion

High Yoba^®^ concentrations led to significantly increased neuromuscular excitation in *Drosophila melanogaster* (Fig. 1a, b and Table 1), offering a basic insight on the physiological effects of *Lactobacillus rhamnosus GG* strain in animals. This was important since motor function in *Drosophila melanogaster* is under the control of the brain [18, 19]. Findings in the study offer a basis for the improved exercise performance following chronic probiotic consumption in mice [20], thus providing evidence that Yoba^®^ once consumed at higher concentrations (above 60,000 CFUs) would lead to increased neuromuscular activation in mammals. In addition, findings in this study justify the therapeutical consumption of probiotics to manage constipation in humans [1, 2, 33] through a modification of gastrointestinal ecology [4, 5, 8, 9]. The study also showed that high Yoba^®^ consumption in *Drosophila melanogaster* led to significantly decreased hydrogen peroxide scavenging activity (Fig. 1c and Table 1). Since increased hydrogen peroxide scavenging activity leads to a reduction in oxidative stress [16], findings in the study support the hypothesis that Yoba^®^ consumption is recommended at low concentrations (1% = 10,000 CFUs) and an excess of these, would negatively affect the brain—gut axis (data not shown).

Low concentrations (≤ 30,000 CFUs) of Yoba^®^ were associated with increased levels of total protein, catalase, and lifespan (Fig. 2a–c). Increased catalase activity would lead to increased oxidative stress resistance [24, 27], leading to an increase in lifespan in *Drosophila melanogaster*. In this study, the emphasis was placed on catalase since this is naturally expressed in *Drosophila melanogaster* in comparison to superoxide dismutase [24] showing that increased catalase activity would increase lifespan, and this was in agreement with previous findings [25, 26]. Catalase has been associated with an increase in resistance to oxidative stress while glutathione reductase increases lifespan at increased levels of oxidative stress, demonstrating the importance of antioxidant enzymes in *Drosophila melanogaster* [28]. Furthermore, lifespan in *Drosophila melanogaster* is affected by dietary protein [23], which would be associated with high Yoba^®^ concentrations in fly food. In the study, this was not investigated, thus offering a window for prospective studies to promote safety on probiotic consumption.

## Limitations

This was a short-lived study, thus a follow-up study to assess chronic effects of Yoba^®^ would offer more insights using both male and female flies since findings in the study are limited to male flies alone. Quantification of the amount of Yoba^®^ ingested, stability after degradation by the gut enzymes and protein—aminoacid composition would offer insights on a correlation between lifespan and concentrations of protein following ingestion of *Lactobacillus rhamnosus* GG. Information on heat shock proteins, cytokines, and neurotransmitter activity would help offer clearer insights on primary inflammatory markers in the brain—gut axis under a Yoba^®^ enhanced diet. Finally, the study didn’t treat *Drosophila melanogaster* for background microbiome, thus findings in the current study offer basic insights on probiotic interactions in body tissues.

## Data Availability

Data files can be accessed at https://figshare.com/s/ca5b72d8c630a8e26367.

## Abbreviations

ANOVA: analysis of variance
CFUs: colony forming units
w/v: weight by volume
